# Survival analysis and classification methods for forest fire size

**DOI:** 10.1371/journal.pone.0189860

**Published:** 2018-01-10

**Authors:** Pier-Olivier Tremblay, Thierry Duchesne, Steven G. Cumming

**Affiliations:** 1 Département de mathématiques et de statistique, Université Laval, Québec, Québec, Canada; 2 Département des sciences du bois et de la forêt, Université Laval, Québec, Québec, Canada; Oregon State University, UNITED STATES

## Abstract

Factors affecting wildland-fire size distribution include weather, fuels, and fire suppression activities. We present a novel application of survival analysis to quantify the effects of these factors on a sample of sizes of lightning-caused fires from Alberta, Canada. Two events were observed for each fire: the size at initial assessment (by the first fire fighters to arrive at the scene) and the size at “being held” (a state when no further increase in size is expected). We developed a statistical classifier to try to predict cases where there will be a growth in fire size (i.e., the size at “being held” exceeds the size at initial assessment). Logistic regression was preferred over two alternative classifiers, with covariates consistent with similar past analyses. We conducted survival analysis on the group of fires exhibiting a size increase. A screening process selected three covariates: an index of fire weather at the day the fire started, the fuel type burning at initial assessment, and a factor for the type and capabilities of the method of initial attack. The Cox proportional hazards model performed better than three accelerated failure time alternatives. Both fire weather and fuel type were highly significant, with effects consistent with known fire behaviour. The effects of initial attack method were not statistically significant, but did suggest a reverse causality that could arise if fire management agencies were to dispatch resources based on a-priori assessment of fire growth potentials. We discuss how a more sophisticated analysis of larger data sets could produce unbiased estimates of fire suppression effect under such circumstances.

## Introduction

Forest fires are important events in many terrestrial ecosystems, including the boreal forests of North America. In many areas where they occur, fire management agencies attempt to control the growth and limit the size of these fires, to protect human lives, infrastructure, and natural resources. The impact of these attempts on fire size has never been fully quantified. Some environmental factors have been shown to affect fire size. For example, several correlative studies have established relationships between fuel type and meteorological indices of fuel moisture content on parameters of fire size distribution [1][2]. Several studies have established correlations between fire management actions and the probabilities of events such as of a fire exceeding the size thresholds established as control targets [3][4] or of a large fire escaping containment [5]. Similar correlations are known for regional indicators such as total annual area burned [2]. These events and indicators are all related to fire size distribution in some way, but none of these studies quantify how fire management affects the size of individual fires. In this study, we present a novel application of survival analysis methods to quantify these effects.

Survival analysis is a kind of regression analysis where the response variables are times to events (e.g. the time from diagnosis of disease to morbidity or death or time from purchase of a device until its first failure). As with survival times, fire sizes are strictly positive random variables with a generally skewed distribution [6], so that the assumptions of normality of residuals are violated under simple analytical techniques such as linear regression. Censoring and truncation are other features typical of time-to-event data [7] that are also exhibited by fire size data. Censoring in this instance arises when a fire is still growing at data collection, here, at the state of Being Held. Truncation occurs because fires are of positive size at first observation. Survival analysis methods allow one to account for these features. Survival analysis methods have recently been applied to fire duration data [8] where the control time, defined as the elapsed time between Initial Attack (IA) and Under Control (Table 1), was related to size at IA (Table 1) and to the prevailing conditions of wind speed and fuel moisture. In studies of fire management and fire ecology, the main interest is in the size of fires, rather than in their duration. However, mathematically, the analysis of time to event data and fire size data are analogous, because of the shared properties of non-negativity and that sequential observations are non-decreasing. Further, a fire size observed at a given time is the time integral of a non-negative growth process up to that time, so the quantities of duration and size are closely related. Survival analysis depends on the survival and hazard functions of the responses which are defined similarly for both duration and size data. Survival analysis of duration data allows the estimation e.g. of the effect of medical treatments on life span, controlling for other factors such as the age of the patient. Similarly, survival analysis of fire size data offers the possibility of estimating the effect of fire management action on fire size while controlling for factors such as fuels or weather. In this way, one could assess the relative effectiveness of alternate control methods, at the level of individual fires, or the aggregate impact of fire management at the population level i.e. the achieved reduction in total area burned relative to natural conditions. In this paper, we present what we believe to be the first such analysis. Specifically, we test for the effect of intervention type (e.g. ground or airborne firefighters, the presence of heavy equipment such as air tankers) and of other management and environmental factors on fire size, using data from boreal forests of northern Alberta, Canada.

**Table 1:** Definition of each variable considered for inclusion in models.

Variable	Defintion
Being Held (BH)	Indicates that with currently committed resources, sufficient suppression action has been taken that the fire is not likely to spread beyond existent or predetermined boundaries under prevailing and forecasted conditions.
Buildup Index (BUI)*	Measure of fuel availability.
Detection	A system for or the act of discovering, locating and reporting wildfires.
Fine Fuel Moisture Code (FFMC)	A numerical rating of the moisture content of litter and other cured fine fuels. This code indicates the relative ease of ignition and flammability of fine fuel.
Fire load*	Number of assessed fires on the same day within the sampled region.
Fire Weather Index (FWI)	A numerical rating of fire intensity that combines ISI and Buildup Index (BUI). It is suitable as a general index of fire danger throughout the forested areas of Canada.
Fuel type	An identifiable association of fuel elements of distinctive species, form, size, arrangement and continuity that will exhibit characteristic fire behaviour under defined burning conditions.
Initial Attack (IA)	The action taken to halt the spread or potential spread of a fire by the first fire fighting force to arrive at the fire.
Initial Spread Index (ISI)	A numerical rating of the expected rate of fire spread. It combines the effect of wind and FFMC on rate of spread but excludes the influence of variable quantities of fuel.
Method	Personnel trained, equipped and deployed to conduct suppression action to halt the spread or potential spread of a wildfire within the first burning period.
Month*	Month of the year the fire was initially attacked (May, June, July, August and September).
Period*	Period of the day initial action began (AM, PM and night).
Response time	The period from receipt of first report of a fire to start of actual fire fighting.
Under Control	Having received sufficient suppression action to ensure no further spread of the fire.

Except as indicated by (*) definitions follow the 2002 glossary of forest fire management terms (Canadian Interagency Forest Fire Centre).

## Materials and methods

### Study area and data

**Fig 1:**
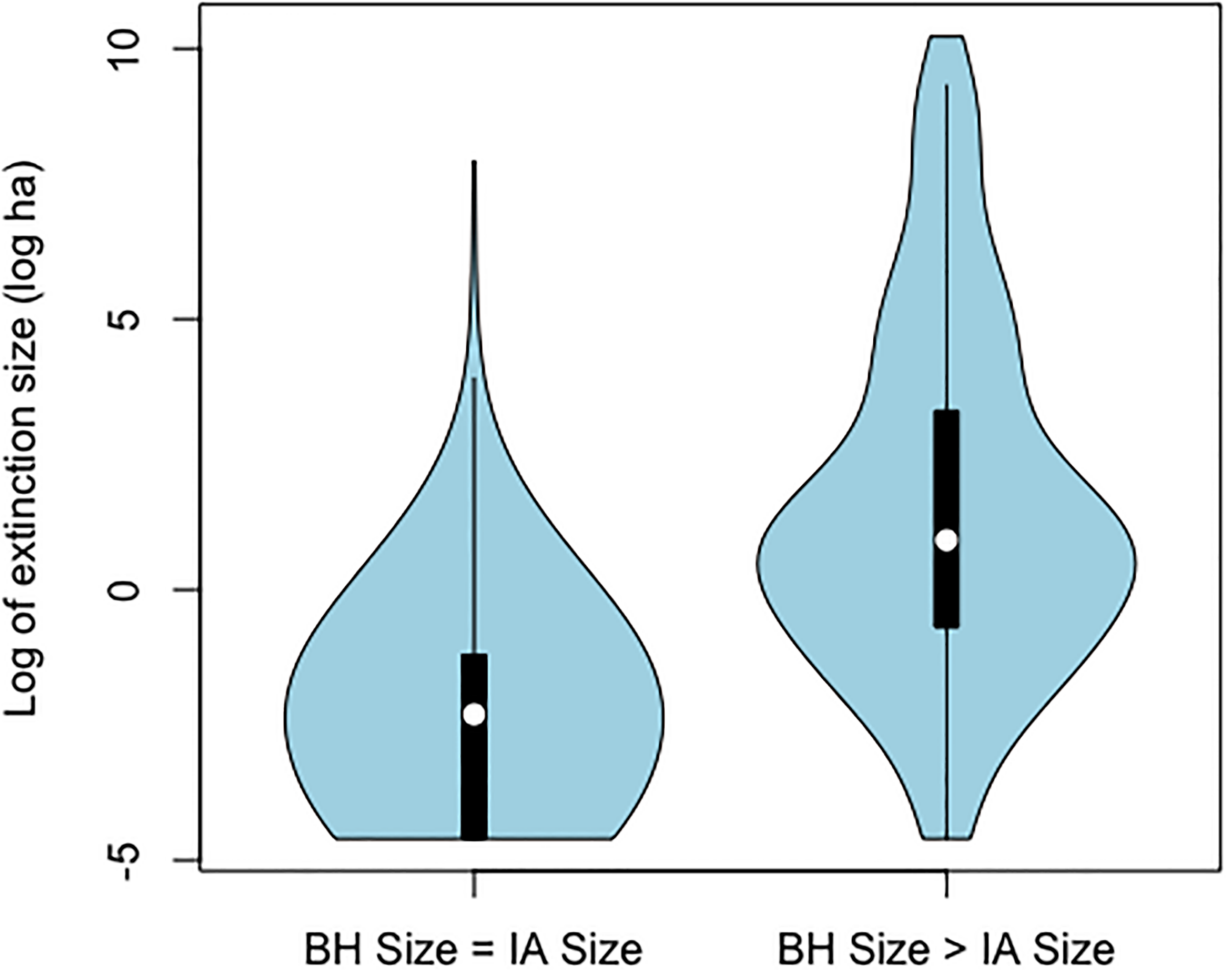
Kernel density of extinction size, given that size at BH was equal to, or greater than, size at IA.

We reanalysed a data set of 960 lightning fires, first used by [3]. These were are all such fires recorded over a seven year period (1996–2002) within a 67,000 km^2^ study region of boreal forest in northeastern Alberta, Canada [3][Fig. 1(a)]. Fire records were selected from the Alberta government’s Historical Wildfire Database [9], with fire weather attributes added by [3]. We used the fire weather variables FWI and ISI (Table 1) because of their relation to fire behaviour [10]. Other variables available at the time of Initial Attack (IA), selected or derived, include the fire sizes at the events of IA and Being Held (BH) (see definitions in Table 1), the Month, period-of-day (Period), fuel type, and fire load at IA, the response time and the methods of intervention (Method) and of initial detection (Detection). We excluded six fires where IA occurred between the hours of 23h00 and 04h00, following [8]. We also excluded 65 fires with data coding errors indicated by negative response times, or a size at IA greater than at BH. A total of 889 fires were retained for analysis. The factor levels for each categorical variable, and their frequencies, are given for these fires in Table 2. The three most common fuel types were C1 (Spruce-lichen Woodland), C2 (Boreal spruce) and M-2 (Boreal mixedwood-green) (See S1, S2, S3 and S4 Figs for distribution of fires among these fuel types). The dominant conifer species in C1 and C2 fuels is black spruce (*Picea marina*). M-2 refers to the characteristic regional mixture of white spruce (*P. glauca*) and trembling aspen (*Populus tremuloides*), after spring leaf-out. The detailed characteristics of these fuel types and illustrative photographs are given in [11]. The most common method of initial attack (Air) refers to helicopter-borne teams of 4 to 7 trained wildland firefighters. “Air tanker” and “Helitanker” refer to fixed wing and rotary wing aircraft, respectively, that are equipped with water tanks that can be poured from above to damp burning fuels or to wet unburned fuels so as to reduce their flammability. “Ground trained” and “Other ground” refer to a variety of predominantly ground-based attack methods. Actions attributed to forest protection officers were assigned to the former class (See S5, S6, S7 and S8 Figs for distribution of fires among these methods).

**Table 2:** Frequencies of factor levels among all 889 fires, for all categorical variables.

Variable	Factor level					Total
Fuel type	C1	C2	M2	Other		889
	61	667	92	69		
Method	Air	Air Tanker	Ground trained	Helitanker	Other ground	889
	615	99	15	25	135	
Month	May	June	July	August	September	889
	108	307	258	201	15	
Detection	Air		Lookout tower	Unplanned		889
	216		488	185		
Period	AM			PM		889
	420			469		

In survival analysis of time-to-event data, event times are strictly larger than the corresponding times of left truncation. By extension, one would expect that sequentially observed sizes (e.g. size at BH) would be strictly larger than the left truncation sizes (e.g. size at IA). This is not always true in the current dataset: sometimes the recorded sizes at IA and BH are the same. There are at least two possible reasons why this may occur for a given fire. Either the fire was already extinguished or quiescent at IA, or the fire size increased slightly but the difference in sizes was not measured accurately. To contribute to the estimation of a continuous size distribution, a fire must register an increase in size [12]. Therefore survival analysis could only be applied to those 260/889 fires that did register such an increase. This raised the question of whether the fires that registered such an increase could be distinguished from those that did not. Accordingly, we conducted a separate classification analysis on the full dataset.

### Classification methods

We defined the classification variable *Y*_*i*_ as the indicator that the *i*^*th*^ fire does not grow, viz,

$$\begin{array}{c} Y_{i}=\{\begin{array}{cc} 0 & \text{if}\;{\text{growth}}_{i}>0\\ 1 & \text{if}\;{\text{growth}}_{i}=0, \end{array} \end{array}$$ (1)

where growth_*i*_ is the difference in size at BH and at IA for the *i*^*th*^ fire. We considered three different classification methods to estimate the odds of having an increase in size (growth_*i*_ > 0) or not (growth_*i*_ = 0) (Eq 1). The methods considered are logistic regression as a generalized linear model (GLM), classification trees, and logistic regression as a generalized additive model (GAM). All models were fit on a training set that contained 2/3 of the dataset and their predictive accuracy was assessed on a validation set consisting of the remaining 1/3 of the data. GLMs were fit using the binomial family with logit link, with a backward variable selection procedure based on significance testing using a variable retention criterion of *p* = 0.05. Classification trees were pruned by using the cross-validation approach described by [13].

All models used the same variable set (see S1 Table), except that the logistic regression models included some square- or logs-transforms of continuous variables. The use and the functional form of covariate transformations were based on an initial GAM analysis, except when the logarithmic transformation was used; the latter was applied to variables that had very large observations that were far from the bulk of the data. We used the area under the Receiver Operating Characteristic (ROC) curves to evaluate model’s predictive power and to determine the best classification method. The ROC curves were interpreted following [14], where values below 0.7 are considered to indicate poor discrimination and values between 0.7 and 0.8 are considered acceptable. All statistical analyses in this paper were performed in R [15]. We used the gam package [16] for generalized additive models, the rpart package [17] for classification trees and the pROC package [18] to obtain the area under ROC curves.

### Survival analysis

We built survival models for those fires that registered a growth in size. We treated IA size as the left truncation “time point” and BH size as the “time point” of the event of interest. We considered two families of models: Cox proportional hazards (PH) and log linear accelerated failure time (AFT) models [12]. We considered three distributional families for the AFT model: Weibull, Lognormal and Loglogistic [12]. We built the models starting from four initial sets of variables, subject to a backward selection procedure. Variables common to each set included FWI, method, fire load, period, month, response time, detection, fuel type, and an interaction term between fire load and method. Two of the four variable sets explored alternate options for including the type of intervention in the model. The other two contrasted alternate ways of accounting for the size at IA. Type of intervention was either a) treated as any other covariate in the backward selection procedure, or b) forced into the model. Because size at IA is already included in the analysis as the left truncation point, it was unclear if we needed to further consider it as a potential covariate. Therefore IA size was either a) the left truncation point and a covariate subjected to the backward selection procedure; or b) the left truncation point only. When treated as a covariate, IA size was log transformed, as above. To variable sets that included log IA size, we added its interactions with FWI, fuel type and method. The choice of interaction terms reflect common knowledge that increasing the number of simultaneous fires limits resource availability, and known relations between management actions and fire size [2–4]. For each model, the covariates subject to exclusion were selected by the backward selection procedure, with exclusion p-value set at 0.05. Cox PH models were fitted with the survival package [19] while AFT models were fitted with the flexsurv package [20].

This process resulted in 16 final models: four Cox PH models and four models for each of the three families of AFT models considered. These models were used as a variable screening procedure to identify a small set of common covariates that were significant in more than one of the Cox or AFT models. The Cox and AFTs models were then re-estimated using only these identified covariates. The best AFT model was then selected by Akaike criteria (AIC). Validation of the final Cox PH model was done using Schoenfeld (cox.zph test) [19], martingale [19] and Cox-Snell residuals [21]. The final choice between the Cox PH model and best AFT model was guided by plots that overlay the cumulative baseline hazard of each model over a nonparametric estimator thereof, as suggested in [12].

## Results

### Classification methods

**Fig 2:**
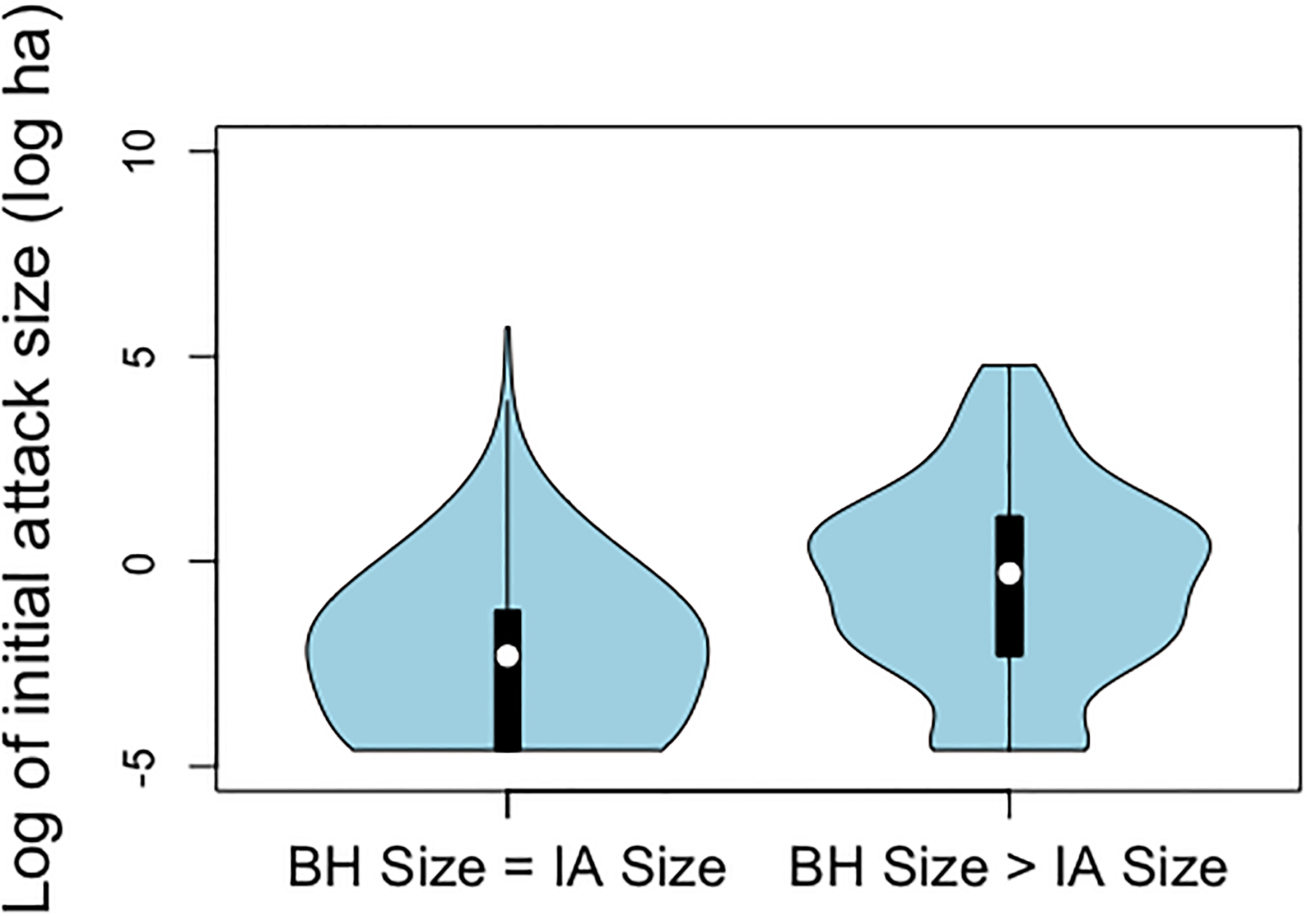
Kernel density of initial attack size, given that size at BH was equal to, or greater than, size at IA.

Our dataset contained 889 lightning fires. Of these, 260 (29%) recorded an increase in size between IA and BH. Median size at IA of these 260 fires was 2.25 ha. The mean size was 643.8ha. The large difference between the mean and median size is due to some large fires, for example the largest fire was 27,490 ha. Of these 260 fires, 25% had a size greater than or equal to 3 ha when IA action began, compared to 3% for the 629 fires that didn’t increase in size [3]. Most of the fires that didn’t register a size increase between IA and BH had final sizes below 1 ha, while most of the remaining fires had final sizes larger than 1 ha (Figs 1 and 2).

Based on ROC scores computed on the validation dataset, logistic regression was more effective than the classification tree in distinguishing the two groups of fires (Table 3). GLM and GAM logistic regression models performed equally well (Table 3). Based on ROC scores, regression models using FWI performed very nearly as well as those using ISI. Given the definitions of these indices, ISI is more dependent on wind speed on the day of the fire, and on the moisture content of the finest fuels. We therefore considered that FWI would be the least temporally variable and hence the better predictor of fire growth conditions over the space of a few days. Accordingly, we use FWI in this and all subsequent models. We report only the GLM model, as being the more interpretable (Table 4). This model fitted to the training dataset (Table 4) shows associations between the probability of growth and the time of day of IA, log IA size, FWI and response time. No model selected fire load, nor were any interaction terms selected. As measured by the area under the ROC curve, the classification accuracy was acceptable (0.75) [14].

**Table 3:** Comparative performance of alternate classifiers on the test data. These models all classified fires into two groups according as at BH was reportedly greater than, or equal to, size at IA.

Classification method	Area under ROC curve	
	FWI	ISI
Logistic regression (GLM)	0.75	0.76
Logistic regression (GAM)	0.75	0.76
Classification tree	0.68	0.70

**Table 4:** Parameter estimates, standard errors (Std. Error) and p-values from the fitted logistic regression (GLM) model.

Variable	Estimate (Beta)	Std. Error	p-value
(Intercept)	0.72	0.26	0.005 **
Period: PM	0.46	0.20	0.022 *
log(IA Size)	-0.39	0.05	<0.001 ***
FWI	-0.04	0.01	0.002 **
log(Response time)	0.09	0.07	0.179
log(Response time)^2^	0.05	0.02	0.014 *

* *p* < 0.05,

** *p* < 0.01,

*** *p* < 0.001

### Survival analysis

We conducted survival analysis on the 260 fires which increased in size after IA. Size at BH was the response variable and size at IA was used as left-truncation. None of the responses were censored. All of our four final Cox PH models had FWI and Fuel type as significant variables. Among the two models where it was available for selection, log IA size was significant in only one, and that model did not include type of intervention. No other variables were retained. However, we added IA method because it was the covariate of greatest interest. Hence, our final models contain FWI, Fuel type, and IA method, or the type of intervention.

**Fig 3:**
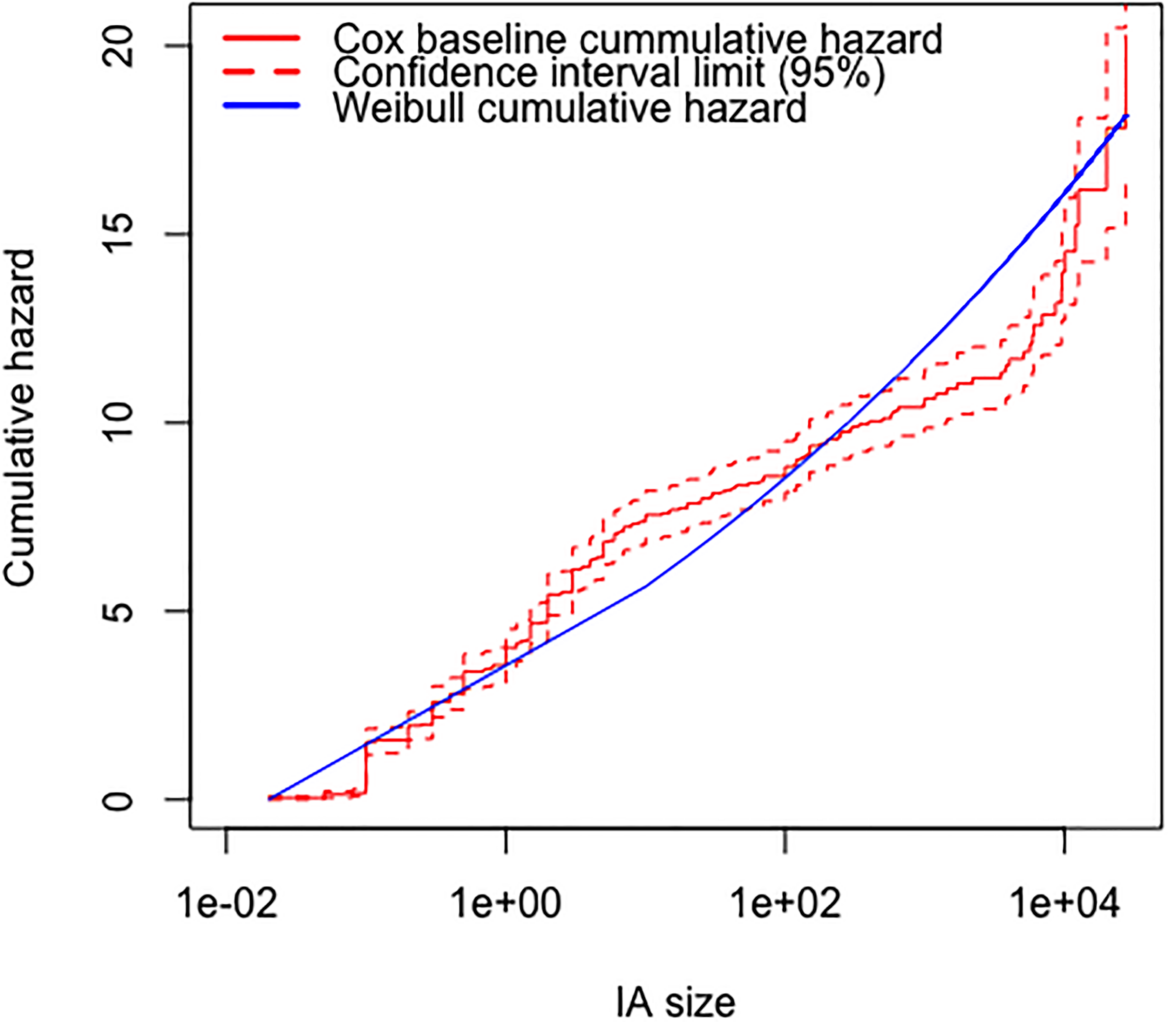
Comparison of Cox PH baseline cumulative hazard and Weibull cumulative hazard. The Weibull cumulative hazard line is often outside the nonparametric 95% (red dashed line) confidence bands for this cumulative hazard function, while the Cox PH baseline cumulative hazard stays within the bands, strongly suggesting that the Cox PH model fits the data better than the Weibull.

**Fig 4:**
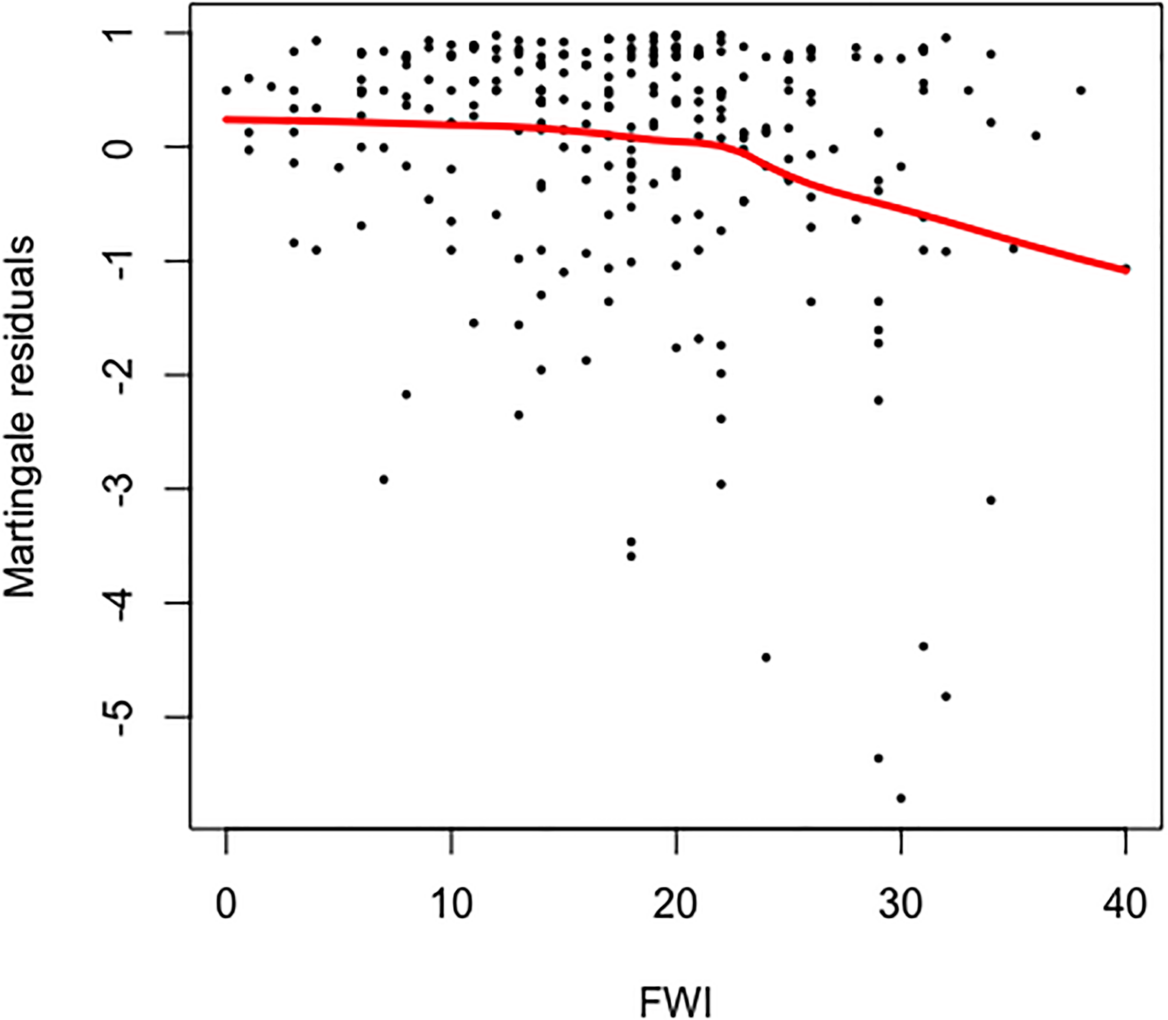
Martingale residuals of Fire Weather Index variable. Locally Weighted Scatterplot Smoothing (LOWESS) (red line) of the martingale residuals shows that they are approximately uncorrelated with mean 0, suggesting that FWI linear functional form is indicated.

**Fig 5:**
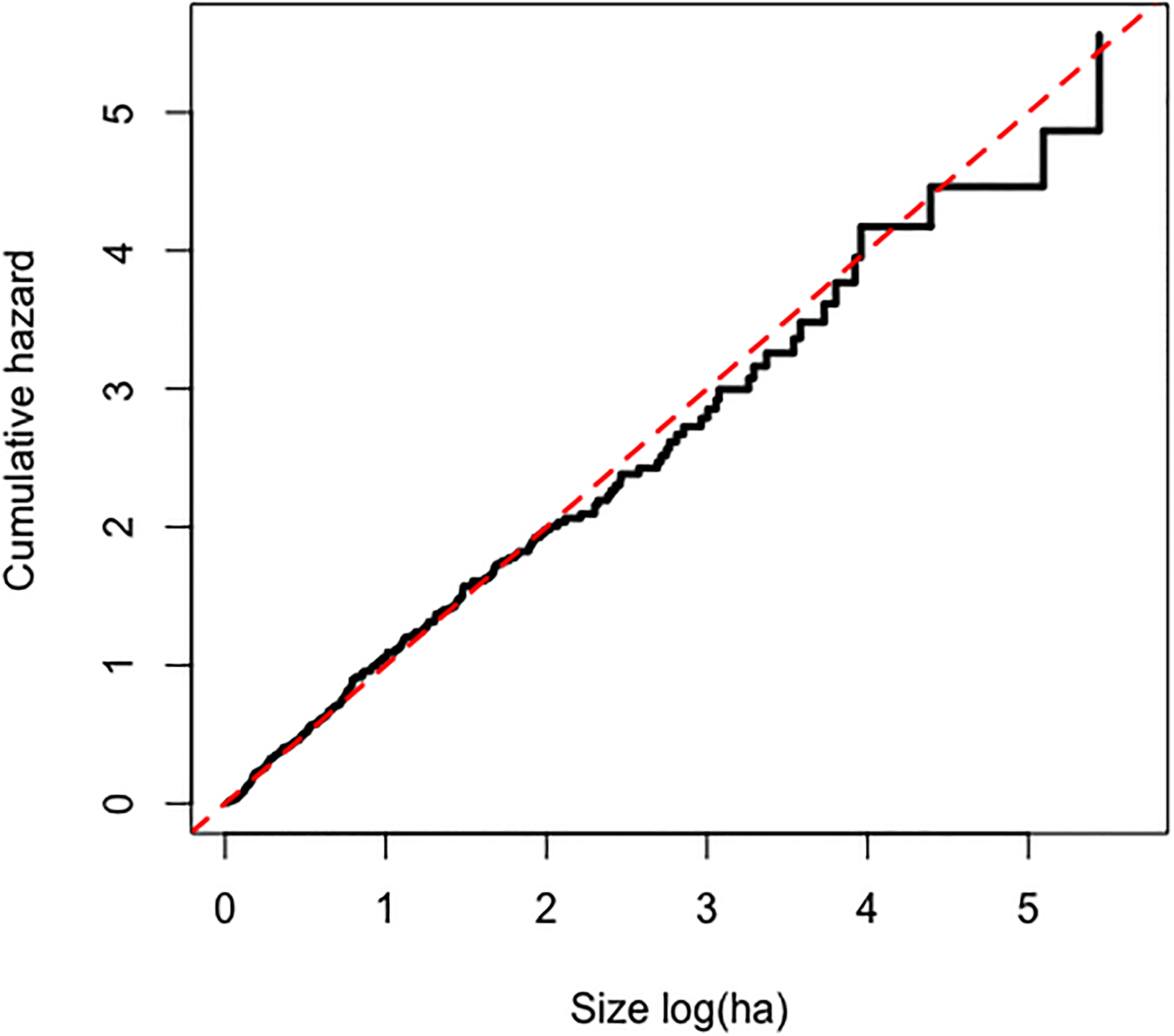
Cox-Snell residuals. The estimated cumulative hazard function of the Cox-Snell residuals (black line) is close to the cumulative hazard of a unit exponential distribution (red dashed line), as expected of a correctly specified model.

**Fig 6:**
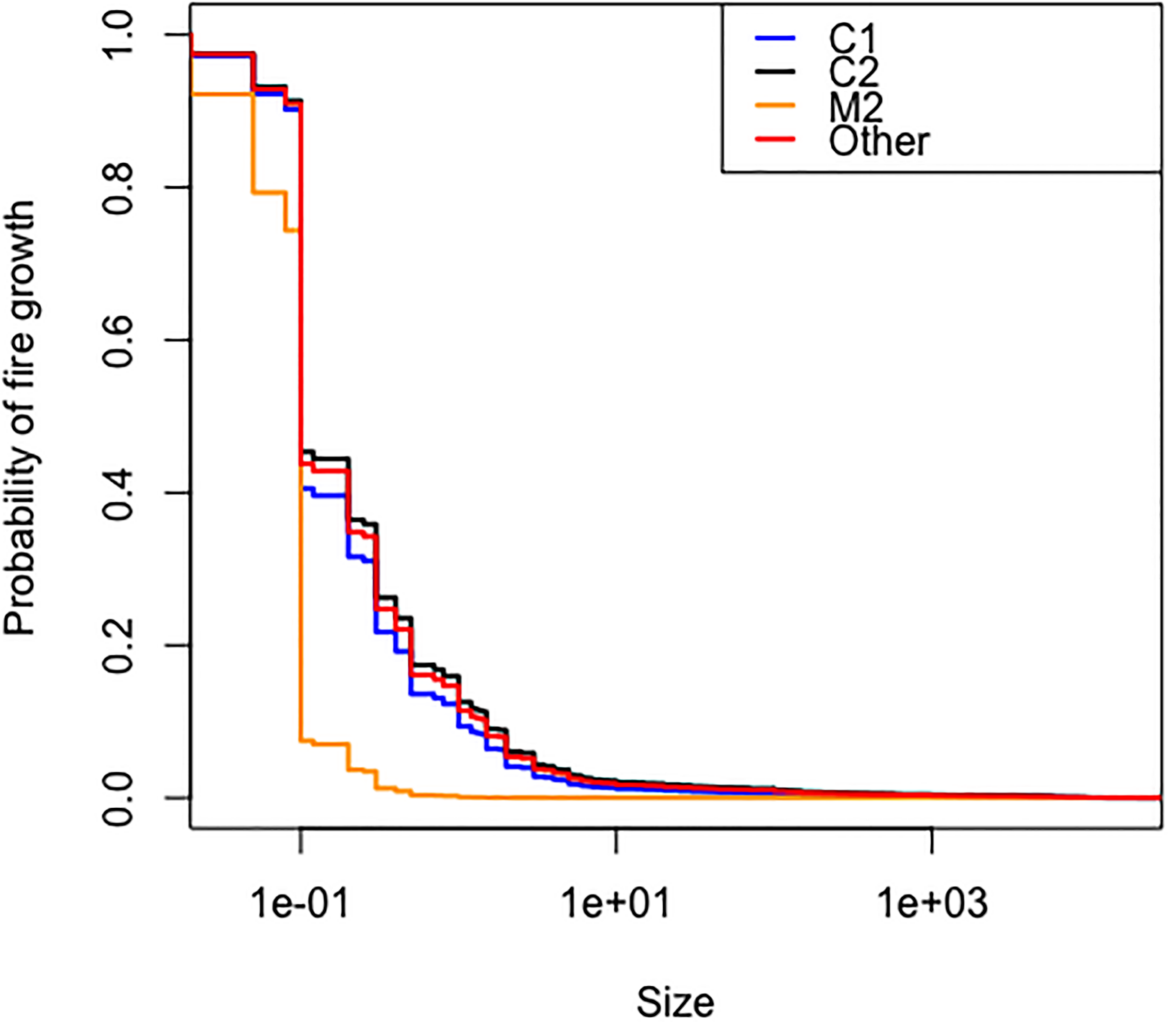
Cox proportional hazard model fitted survival curves for all levels of fuel type under air attack and median FWI. Levels of Fuel Type are represented by C1 (blue line), C2 (black line), M2 (orange line) and Other fuel type (red line).

**Fig 7:**
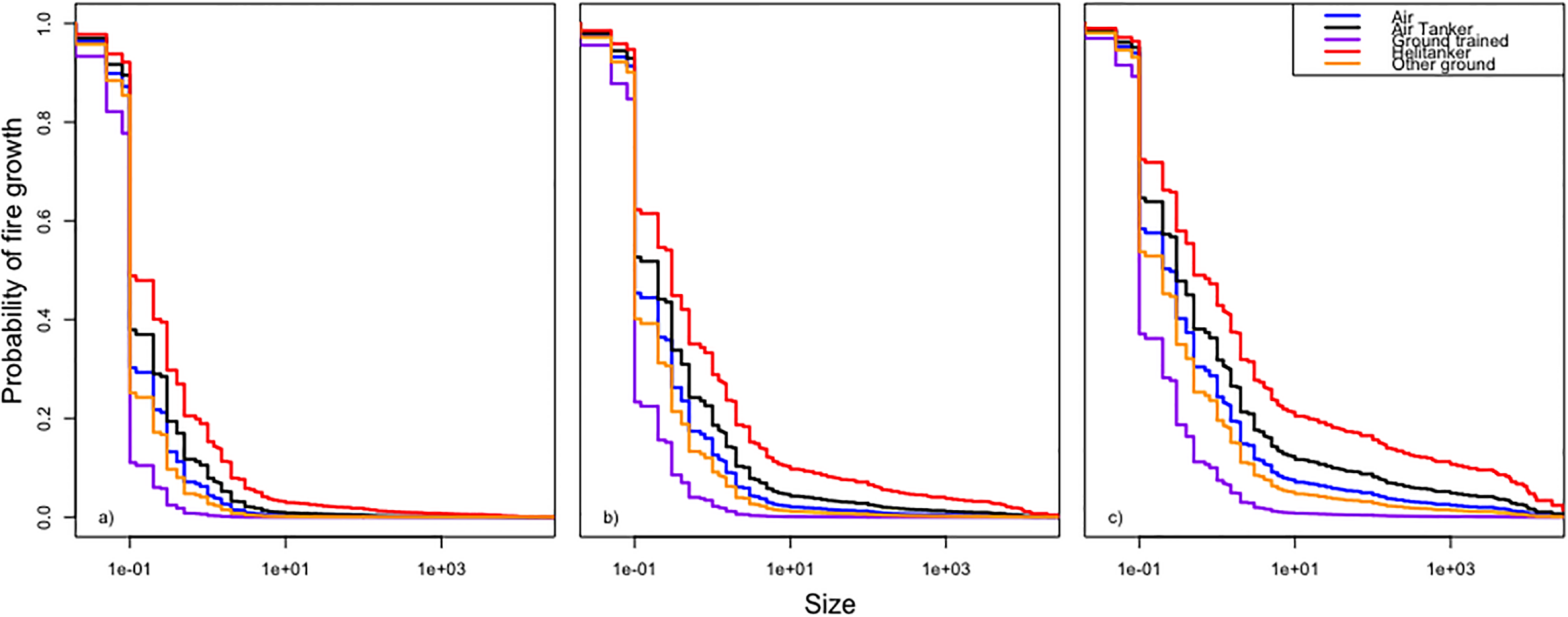
Cox proportional hazard model fitted survival curves for all levels of IA method under fuel type C2 and contrasting levels of FWI. Levels of Method are represented by Air (blue line), Air tanker (black line), Ground trained (purple line), Helitanker (red line) and Other ground crews (orange line). FWI values are respectively the 5th (a) FWI = 4), 50th (b) FWI = 18) and 95th (c) FWI = 31) percentile.

The Weibull distribution was the best of the three parametric models according to the AIC criterion (Table 5). The cumulative baseline hazard function of the Cox PH model provides a better fit to the data than its counterpart from the Weibull AFT model (Fig 3). The model validation based on residuals suggested that the overall fit of the Cox PH model was good. The hypothesis of proportional hazards could not be rejected (p = 0.079 for the cox.zph test based on Schoenfeld residuals). The distributions of martingale residuals (Fig 4) and Cox-Snell residuals (Fig 5) also supported the Cox PH model. Accordingly, we adopt this model over the parametric alternatives (Table 6). The significant variables were FWI and Fuel type (Table 7). FWI was highly significant with a negative parameter estimate (Table 6). High values of FWI are associated with a lowered risk of a fire being held, i.e., increasing FWI is associated with larger sizes at BH (Table 6). Within Fuel type, only M2 (Boreal mixedwood) was significant with a positive parameter estimate (Table 6). Relative to the base case (C1), M2 is associated with a higher risk of a fire being held, i.e., with smaller sizes at BH. The other fuel types did not differ from the C1 type in this respect (Fig 6). IA Method was not significant overall (Table 7) or for any contrast level (Table 6). Nevertheless, the method-specific parameter estimates suggest that different attack methods may be associated with different distributions of fire sizes (Fig 7).

**Table 5:** AIC scores of three alternate forms of parametric survival model.

Functional form	AIC
Weibull	1713.30
Lognormal	1731.03
Loglogistic	1906.94

**Table 6:** Parameter estimates, standard errors (Std. Error) and p-values from the fitted Cox proportional hazards model.

Variable	Estimate (Beta)	Std. Error	p-value
FWI	-0.03	0.01	<0.001 ***
Method:Air tanker	-0.21	0.20	0.283
Method:Ground	0.61	0.60	0.306
Method:Helitanker	-0.51	0.31	0.105
Method:Other	0.14	0.18	0.426
Fuel type:C2	-0.13	0.27	0.632
Fuel type:M2	1.05	0.39	0.007 **
Fuel type:Other	-0.09	0.37	0.813

* *p* < 0.05,

** *p* < 0.01,

*** *p* < 0.001

**Table 7:** Likelihood-ratio chi-square, degrees of freedom and p-values from the fitted Cox proportional hazards model analysis of deviance (Type II tests).

Variable	LR Chisq	Df	p-value
FWI	13.68	1	<0.001 ***
Method	6.15	4	0.188
Fuel type	12.63	3	0.006 **

* *p* < 0.05,

** *p* < 0.01,

*** *p* < 0.001

## Discussion

Most (629/889) fires in our final data set showed no recorded increase in size after initial assessment. Survival analysis could not be applied to these fires. To more completely model the fire size data, we employed a two stage analysis. First we used logistic regression to classify our sample into groups which did, or did not, exhibit such size growth. We then applied survival analysis to the former group. We can suggest two possible reasons for membership in the latter group. A fire that was growing very slowly at IA might have exhibited only very slight size growth before extinction, in which case recorded non-growth is a form of measurement error. It’s also possible that some of such fires were already extinguished at the time of IA. This is supported by our classification model, which shows the probability of the event “no growth after IA” was decreased with fire size at IA and with FWI, and thus was associated with small fires under moderate or low burning conditions. In this regard, most of the fires that didn’t increase in size were extinguished below Arienti et al’s containment size threshold of 3 ha (≈1.1 log ha), as opposed to fires that did increase (Fig 1). Furthermore, our results are consistent with their model of containment failure probability [3]. Size of a fire at IA as well as fire weather have a significant association with fire size growth.

We have demonstrated that survival analysis methods can be applied to fire size data, specifically to study the effects of fire suppression on fire size. Survival analysis had already been applied to duration [8], but from a management perspective, area burnt is more relevant. Residual checks and the fact that the Weibull AFT was the best among the parametric models considered suggest that the proportional hazards assumption is reasonable in our context. The Cox Proportional Hazards model was better supported than three parametric AFT alternatives, according to standard model selection criteria and residual checks. It might be the case that other classes of parametric survival models would better fit the data if different distributional families of distributions were supported, such as the truncated Pareto [1] or tapered Pareto [22].

We found an association between Fire Weather Index (FWI) and fire size. The strongly significant negative coefficient for the FWI variable shows that under conditions of high flammability due to low fuel moisture and/or high wind speeds, fires are more likely to become large. This is consistent with associations between related indices and fire duration [8] and of the probability of achieving control targets [3]. We also found an association between fire size and the type of vegetation in which the fire was burning at first action. Fires in M2 fuels tended to be smaller than fires in the base fuel type (C1), and than in other fuel types according to the Cox survival curves Fig 6. This finding is consistent with the differences in burn rate amongst vegetation types in this study region [23].

We also found some indication of an association between type of intervention and fire size, but not in the direction expected. Although the results were not significant at the 0.05 level, the estimated coefficients suggested that fires attacked with the aid of airborne water tanks, especially helitankers, grew larger than those attacked by other methods. The raw data also showed such tendency (S5 Fig), and the difference among group sizes was significant (Kruskal-Wallis $\chi_{4}^{2}=15.22$, *p* = 0.0043). The direction of this possible association is opposite to the expected causal effect. Attacking a fire with a more aggressive method should not tend to increase fire growth relative to a less aggressive method. Such an association could arise, though, due to what is known in the medical literature as “confounding by indication”: When the most radical treatments are given to the sickest patients [24], the associated mortality is likely to be high, even if the treatments are effective in prolonging life. We suspect a similar effect may be operating in our study. Fire weather conditions, both present and forecast, and the probable fuel type, are known to the fire management agencies who dispatch resources to fires. These decisions are based on many factors such as permanent IA base location, number and type of resources available at the time, resource deployment between among facilities, etc. Expected growth rate of a fire is influenced by the human and material resources assigned to the fire and also the quickness of detection and of response by an IA crew [25]. Air tankers and helitankers are scarce resources, so one might expect that they are dispatched selectively to the fastest growing fires or to those that pose the greatest risk. This is consistent with our findings by survival analysis, and with the distribution of size at IA as a function of the attack method. For fires that grow after IA, the distribution of the sizes at IA differs significantly among attack methods (S8 Fig, Kruskal-Wallis test, *p* = 0.030). Based on these post-hoc findings, we conclude that there is some reason to suspect confounding by indication. This suggests that the analytical methods of causal inference could be applied to uncover the true effects of fire suppression [26]. A sample of fires designed so that various treatments have been applied to fires of various IA sizes would be required for this. Moreover, including fire weather data as fire-size or time-varying covariates might better help disentangle the confounding than summarizing the weather as a single time-fixed covariate (e.g., our FWI). Much larger datasets than what we used here could be assembled from fire management archives, which would make it possible to apply these more powerful methods. We note that the covariate of fire load, designed to measure the level of activity of the fire management system at the time of each fire, was not significant. One might expect an interaction between attack method and load, as presumably fire management resources become scarcer when more fires are being fought at the same time, but we did not consider our sample size sufficient to explore an interaction with a multi-level factor. Again, larger sample sizes and more refined covariates might reveal the true effect of daily fire load. The potential size of the non-significant effects of IA method (Fig 7) appear to us large enough to warrant further investigation.

We found that survival analysis methods can unambiguously identify factors such as fuels and fire weather which are not subject to confounding by indication. In health sciences applications, more data intensive survival analysis methods, such as causal inference, can be used to estimate the consequences of medical treatments in terms of years of life saved among a population [27][28], even in the presence of confounding. As noted above, it may be possible to assemble fire size datasets suitable to these methods. With the advent of remote-sensed fire size data obtainable at daily intervals, more informative longitudinal fire growth analysis may also become possible. We hope the present work will encourage further work to ultimately quantify the effectiveness of modern fire management in terms of the area preserved from burning [4].

## Supporting information

S1 Fig Distribution of being held size among each fuel type for all fires. (TIF)

S2 Fig Distribution of being held size among each fuel type given that a fire has grown between IA and BH. (TIF)

S3 Fig Distribution of initial attack size among each fuel type for all fires. (TIF)

S4 Fig Distribution of initial attack size among each fuel type given that a fire has grown between IA and BH. (TIF)

S5 Fig Distribution of being held size among each method of intervention for all fires. (TIF)

S6 Fig Distribution of being held size among each method of intervention given that a fire has grown between IA and BH. (TIF)

S7 Fig Distribution of initial attack size among each method of intervention for all fires. (TIF)

S8 Fig Distribution of initial attack size among each method of intervention given that a fire has grown between IA and BH. (TIF)

S1 Table List of variables available at the beginning of variable selection for classification (except interaction terms) procedure and survival analysis. (TEX)
