# Supplementary figures and images for: Survival analysis and classification methods for forest fire size
Source: PLoS One. 2018 Jan 10;13(1):e0189860. doi: 10.1371/journal.pone.0189860 (PMC5761846; doi:10.1371/journal.pone.0189860)

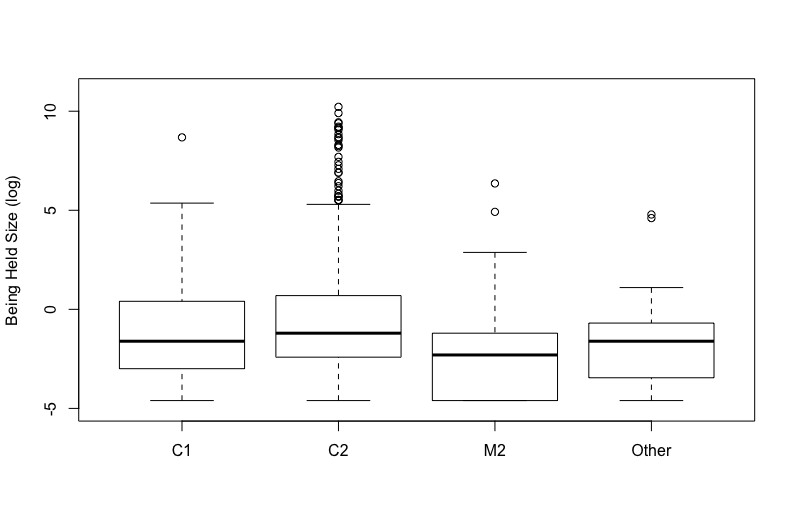

Supplement: S1 Fig — (TIF) [file pone.0189860.s001.tif]

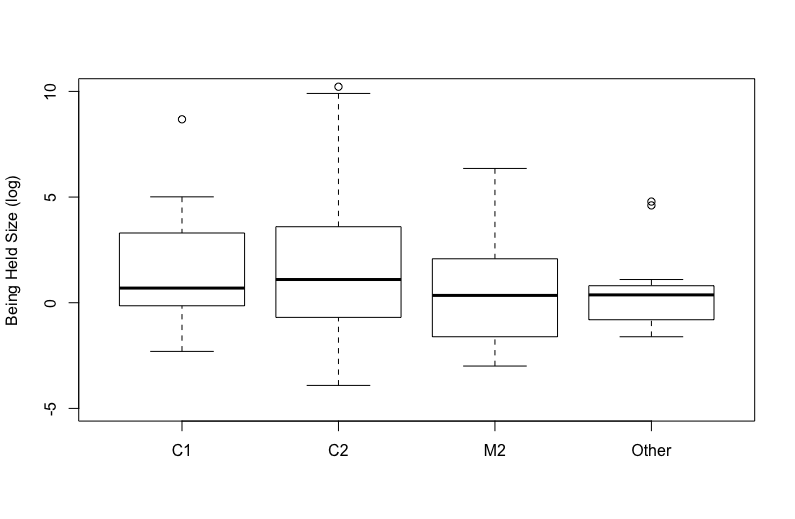

Supplement: S2 Fig — (TIF) [file pone.0189860.s002.tif]

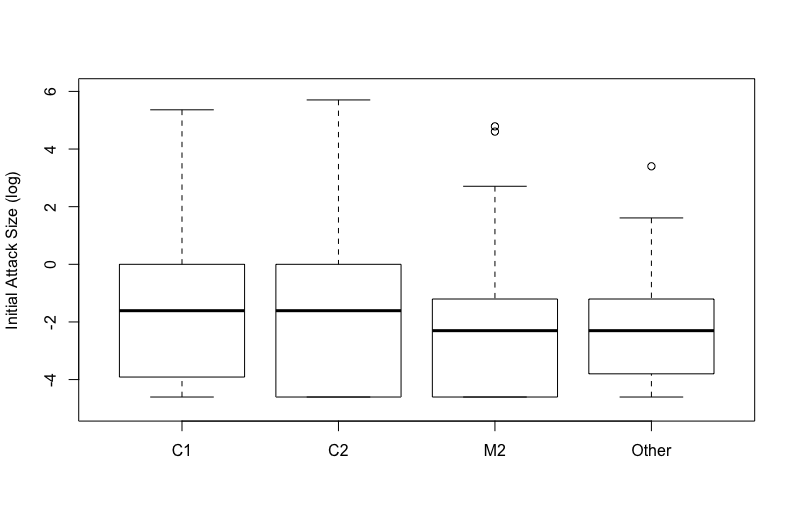

Supplement: S3 Fig — (TIF) [file pone.0189860.s003.tif]

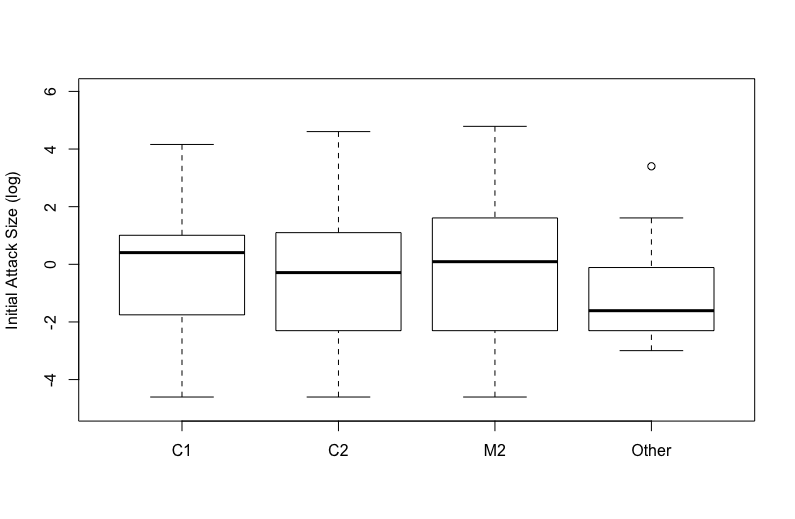

Supplement: S4 Fig — (TIF) [file pone.0189860.s004.tif]

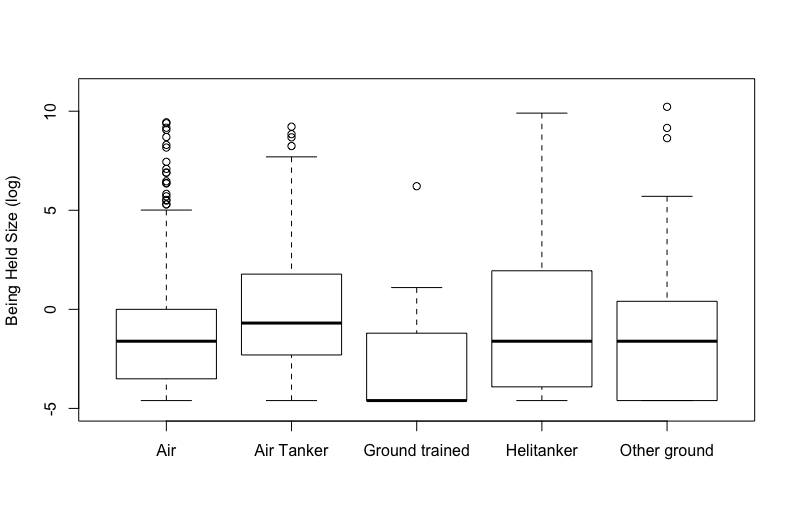

Supplement: S5 Fig — (TIF) [file pone.0189860.s005.tif]

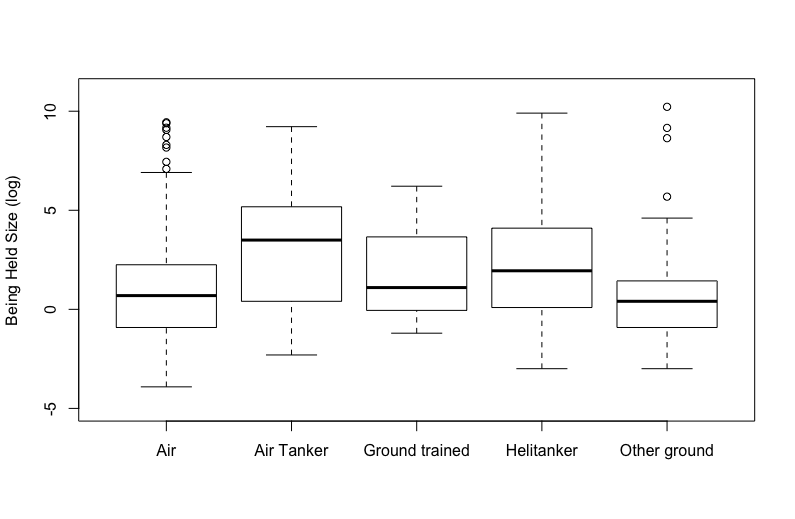

Supplement: S6 Fig — (TIF) [file pone.0189860.s006.tif]

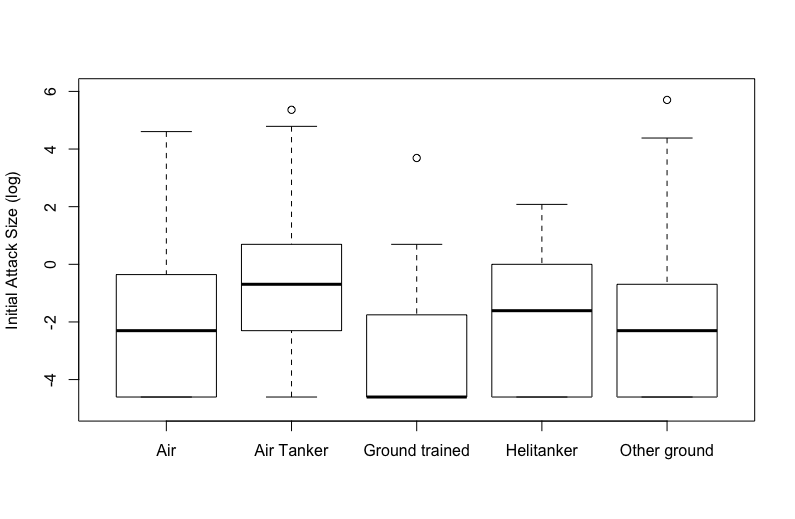

Supplement: S7 Fig — (TIF) [file pone.0189860.s007.tif]

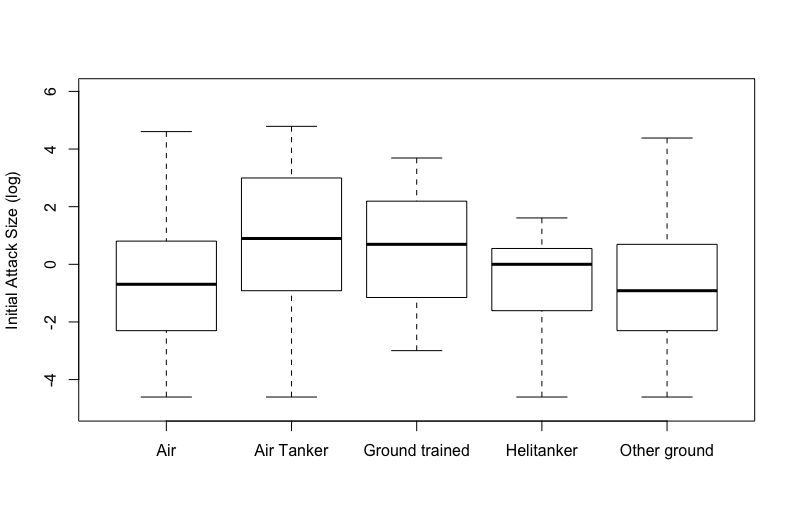

Supplement: S8 Fig — (TIF) [file pone.0189860.s008.tif]
